# Comparative Study of Water and Milk Kefir Grains as Biopolymeric Adsorbents for Copper(II) and Arsenic(V) Removal from Aqueous Solutions

**DOI:** 10.3390/polym16233340

**Published:** 2024-11-28

**Authors:** Balázs György, Marek Bujdoš, Hana Vojtková, Pavel Diviš, Michal Slaný, Peter Matúš, Martin Urík

**Affiliations:** 1 Institute of Laboratory Research on Geomaterials, Faculty of Natural Sciences, Comenius University in Bratislava, Mlynská dolina, Ilkovičova 6, 842 15 Bratislava, Slovakia; gyorgy7@uniba.sk (B.G.); marek.bujdos@uniba.sk (M.B.); martin.urik@uniba.sk (M.U.); 2 Department of Environmental Engineering, Faculty of Mining and Geology, VSB—Technical University of Ostrava, 17. listopadu 2172/15, 708 00 Ostrava, Czech Republic; hana.vojtkova@vsb.cz; 3 Institute of Food Science and Biotechnology, Faculty of Chemistry, Brno University of Technology, Purkyňova 464/118, Královo Pole, 612 00 Brno, Czech Republic; divis@fch.vut.cz; 4 Department of Materials Engineering and Physics, Faculty of Civil Engineering, Slovak University of Technology, Radlinského 11, 810 05 Bratislava, Slovakia; michal.slany@stuba.sk; 5 Department of Materials Engineering and Chemistry, Faculty of Civil Engineering, Czech Technical University in Prague, Thákurova 7, 166 29 Prague, Czech Republic

**Keywords:** biopolymer, biosorption, heavy metals, polysaccharide, remediation, waste biomass

## Abstract

This study investigates the biosorption capabilities of kefir grains, a polysaccharide-based byproduct of the fermentation process, for removing copper(II) and arsenic(V) from contaminated water. Unlike traditional heavy-metal removal methods, which are typically expensive and involve environmentally harmful chemicals, biopolymeric materials such as kefir grains provide a sustainable and cost-effective alternative for adsorbing hazardous inorganic pollutants from aqueous solutions. Our experimental results revealed significant differences in the sorption capacities of two types of kefir grains. Grains of milk kefir outperformed water kefir, particularly in copper(II) removal, achieving up to 95% efficiency at low copper concentrations (0.16 mmol·L^−1^) and demonstrating a maximum sorption capacity of 49 µmol·g^−1^. In contrast, water kefir grains achieved only 35.5% maximum removal efficiency and exhibited lower sorption capacity. For arsenic(V) removal, milk kefir grains also showed superior performance, removing up to 56% of arsenic in diluted solution with experimental sorption capacities reaching up to 20 µmol·g^−1^, whereas water kefir grains achieved a maximum removal efficiency of 34.5%. However, these findings also suggest that while kefir grains show potential as low-cost biosorbents, further modifications are needed to enhance their competitiveness for large-scale water treatment applications.

## 1. Introduction

Biosorption using waste biological materials has gained significant attention in recent years due to their versatile applications in environmental remediation, particularly in the removal of toxic heavy metals, metalloids, and other pollutants from contaminated water sources [1]. Traditional methods for heavy-metal removal, such as chemical precipitation, ion exchange, membrane filtration, and electroplating, often involve high operational costs and the use of environmentally harmful chemicals. In contrast, biosorbents derived from natural and waste materials offer a more sustainable, cost-effective, and eco-friendly alternative to conventional approaches for mitigating heavy-metal contamination in water [2].

To highlight the emerging potential of biopolymeric waste products from the food industry in the bioremediation of heavy-metal-contaminated waters, this study investigates the application of kefir grains, a byproduct of the fermentation process in beverage production [3], as biosorbents. Kefir grains, traditionally associated with the fermentation of dairy and water-based beverages, have recently been considered for the removal of waterborne contaminants, particularly organic toxins [4]. However, their use as biosorbents for inorganic pollutants, such as heavy metals, remains largely underexplored [5]. Therefore, this research specifically focuses on evaluating the sorptive capabilities of both water and milk kefir grains to determine their effectiveness in adsorbing copper(II) and arsenic(V)—two hazardous inorganic contaminants frequently found in industrial effluents and drinking water supplies [6].

Copper(II), though a necessary micronutrient in trace amounts, can become highly toxic when released in excess into the environment through industrial processes, leading to severe health risks for humans, plants, and aquatic ecosystems [7]. Similarly, arsenic(V), which is often found in industrial waste and agricultural runoff, is a major public health concern, poisoning millions globally through contaminated drinking water [8].

Given the high toxicity of these elements, effective removal strategies are crucial for protecting public health and preserving wildlife and aquatic ecosystems. By comparing the sorptive performance of water and milk kefir grains in the removal of copper(II) and arsenic(V), we aim to highlight the feasibility of these natural materials as effective, low-cost alternatives for remediating contaminated water. Ultimately, this study not only highlights the potential of kefir grains as low-cost biosorbents for heavy-metal removal but also sets the stage for future research into modified biosorbents and their broader applications in sustainable water treatment technologies.

## 2. Materials and Methods

### 2.1. Kefir Grain Preparation

Kefir grains were prepared through static cultivation using a commercially obtained inoculum. Water kefir was cultivated in a 1 L solution containing 100 g of sucrose and 200 g of lemon slices, while milk kefir was cultivated in 1 L of commercially available milk. After inoculation, it has been incubated up to 4 days at room temperature. Subsequently, the resulting biomass, consisting of microbial cells and exopolymers, was separated from the solution by filtration, washed with great amounts of distilled water, and used in this form in sorption experiments.

### 2.2. Sorption Experiments

All sorption experiments were conducted at a temperature of 25 °C in sealable 50 mL sterile plastic centrifuge tubes. In each, the 10 g of wet biomasses in the form of grains from water or milk kefir was suspended with 20 mL of an aqueous solution of selected potentially hazardous metals, including arsenic(V) and copper(II), so that the initial concentration of the analyte ranged from 0.14 to 4.2 mmol·L^−1^. The pH of the solutions was adjusted to 6.0 using diluted HCl or NaOH solutions.

For the preparation of stock solutions of arsenic(V) and copper(II), the reagents of p.a. quality were used: Na_2_HAsO_4_·7H_2_O (Sigma-Aldrich, St. Galen, Switzerland) and CuSO_4_·5H_2_O (Centralchem, Bratislava, Slovakia).

Kinetic experiments were conducted to evaluate the sorption rate, examining the effect of contact time on the sorption capacities of kefir grains over a period of 240 min. This duration provided sufficient data to calculate rate constants and offered insights into the sorption mechanism. However, for the isotherm studies, a contact time of 100 h was selected to ensure equilibrium in the sorption system. To ensure homogeneous interaction of the selected analytes with the surfaces of the water or milk kefir grains, the plastic tubes with the sample suspensions were placed on an overhead shaker (MultiBio RS-24; Biosan, Riga, Latvia) at a rotation speed of 45 rpm. Isotherm studies were conducted in triplicate, whereas kinetic experiments were performed in duplicate.

After the required contact time, the samples were centrifuged at 10,000× *g* for 10 min (centrifuge 5804R; Eppendorf, Hamburg, Germany) to separate the biosorbent from the remaining solution. The obtained solution was analyzed for residual concentration of the selected analytes using atomic absorption spectroscopy [9,10,11]. The residual biomass was dried to a constant weight at 85 °C, and the measurements were used in the following statistical procedures.

### 2.3. Analytical and Statistical Procedures

To determine the concentration of arsenic and copper in collected aqueous solutions (filtrates and stock solutions), a flame atomic absorption spectrometer (PerkinElmer 1100, Waltham, MA, USA) with an air-acetylene flame (air flow rate 8.0 L·min^−1^, acetylene flow rate 2.5 L·min^−1^) was utilized. The limits of quantification for arsenic and copper for this method were 0.5 mg L^−1^ and 0.005 mg L^−1^, respectively. Calibration solutions were prepared from CertiPUR ICP 1000 mg·L^−1^ single-element standard solutions (Merck, Darmstadt, Germany).

To evaluate the surface morphology of the biomasses, scanning electron microscopy (SEM) was performed using a Carl Zeiss EVO 40 HV operated at 20 kV with a Bruker energy-dispersive X-ray (EDX) silicon drift detector.

The IR spectra of the kefir grains were measured on a Nicolet 6700 Fourier transform infrared (FTIR) spectrometer (Thermo Scientific, Waltham, MA, USA). For the MIR region (4000–400 cm^−1^), 64 scans were used, and the spectrometer was equipped with a DTGS detector, an IR source, and a KBr beam splitter. The KBr pellet preparation technique was used to prepare the samples, where 1 mg of the sample was mixed with 200 mg of KBr.

The (equilibrium) sorption capacity of the waste-biomass-based sorbent (*S_eq_*) was calculated using Equation (1):

(1) $$S_{eq}=\frac{\left(C_{0}-C_{eq}\right)V}{m}$$

where *C*_0_ and *C_eq_* refer to the initial and equilibrium concentrations (mmol·L^−1^) of the adsorbate in the suspension, respectively. *V* and *m* indicate the volume of the solution and the dry weight of the sorbent, respectively. For the calculation of the minute sorption capacity (*S_t_*) at reaction time *t* (min), the same formula was used, except that the equilibrium concentration has been substituted for the minute concentration of the adsorbate in the solution (*C_t_*).

The calculated sorption capacities at different initial arsenic(V) and copper(II) concentrations were fitted by the standard sorption isotherms, showcased in Table 1, to evaluate the sorption performance of the water and milk kefir grains. For the kinetic modelling, pseudo-first-order and the pseudo-second-order kinetics were employed, listed in Table 2. All statistical and data analyses regarding the sorption experiments were conducted using the Analysis ToolPak version 2.0.0.0 and Solver version 3.0.0.1 add-in programs (Frontline Systems, Incline Village, NV, USA) in Microsoft 365 Excel version 2402 (Microsoft, Redmond, WA, USA).

To assess the appropriateness of each kinetic and isothermal model for the experimental data, Akaike weights (*w_i_*) were utilized as follows:

(2) $$w_{i}=\frac{exp\left[\frac{1}{2}({AIC}_{min}-{AIC}_{i})\right]}{\sum exp\left[\frac{1}{2}({AIC}_{min}-{AIC}_{i})\right]}$$

Here, *AIC* represents Akaike’s information criterion. *AIC*_min_ signifies the lowest *AIC* value among the models, and *AIC*_i_ is the *AIC* value of the specific model *i*. Akaike’s information criterion serves as an estimated measure of the quality of available models for a particular dataset, making it an ideal method for model selection [12]. This approach is advantageous as it considers the number of model parameters (*k*_p_), the number of data points (*n*), and the statistical parameter, which, in our case, is the residual sum of squares (RSS) of the respective model:

(3) $$AIC=2k_{p}+n\ln\left(RSS/n\right)$$

## 3. Results

### 3.1. Structural and Morphological Characterization of the Kefir Grains

**Figure 1:**
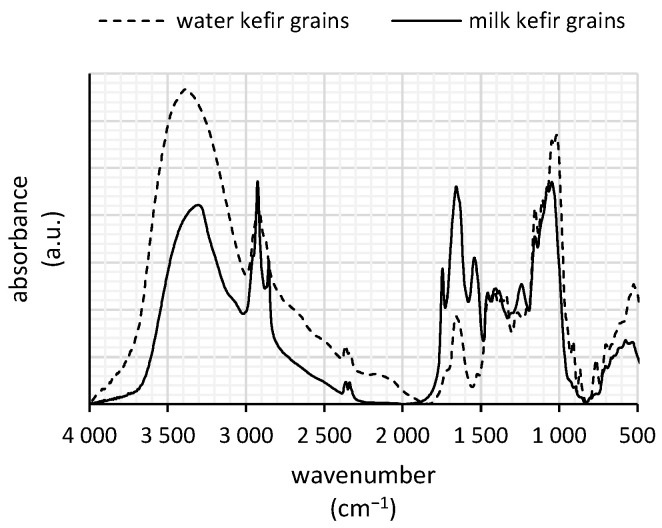
FTIR spectra of water and milk kefir grains.

FTIR analysis was conducted (Figure 1) to identify the main functional groups associated with the polysaccharides and other organic components in water and milk kefir grains. As polysaccharides are expected to be the predominant components in both biomasses, a broad band observed around 3350 cm^−1^ can be attributed to the stretching of hydroxyl groups in carbohydrate structures or O–H stretching vibrations associated with the water content. Additionally, the prominent fingerprint region between 1200 and 900 cm^−1^ suggests the presence of pyranose rings and associated side groups, evidenced by C–O–C stretching vibrations and C–O–H bending vibrations in carbohydrate structures [13]. The strong absorbance at this region indicates glucose and galactose units in milk kefir grains [14], while glucose units of dextran are prominent in water kefir grains [15].

The 3100–2800 cm^−1^ region is dominated by asymmetric and symmetric C–H stretching vibrations, with strong peaks at around 2925 and 2854 cm^−1^, respectively, attributed to aliphatic CH_2_ groups from fatty acids in membrane amphiphiles [16]. For milk kefir grains, these regions can also be linked to a carbonyl stretch near 1745 cm^−1^ suggesting the presence of milk fatty acid remnants in the biomass of milk kefir grains [17].

Notable differences between water and milk kefir grains are observed in the 1800–1200 cm^−1^ range, likely due to a higher content of proteins in milk kefir and their interpolymeric interactions with polysaccharides [14]. The 1800–1500 cm^−1^ region generally reflects the amide I and amide II bands associated with peptides and proteins. Both kefir grains display a distinct band around 1655 cm^−1^, likely corresponding to carbonyl (C=O) stretching in peptides [18]. Additionally, a strong N–H vibration peak at 1540 cm^−1^ is unique to milk kefir grains; this peak, along with the band near 1655 cm^−1^, may be linked to amide linkages involving aminosugars within the polysaccharide structure [19].

**Figure 2:**
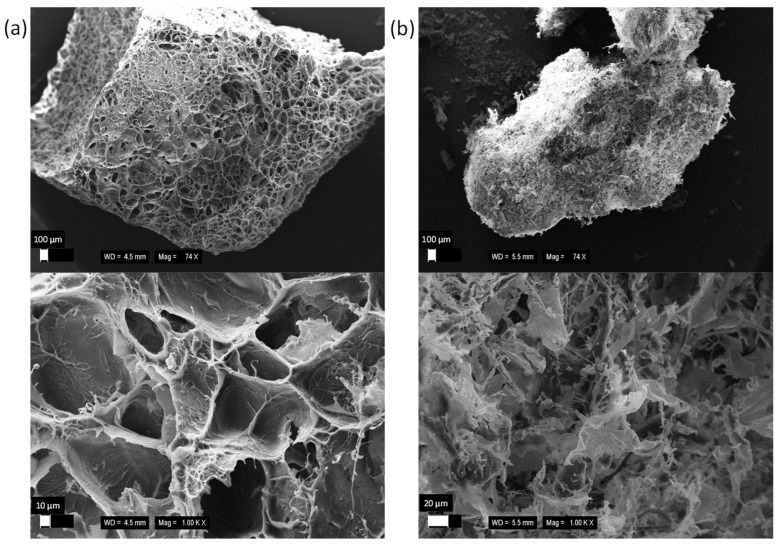
Scanning electron micrograph of (**a**) water and (**b**) milk kefir grains.

The external microstructure of lyophilized kefir grains was examined using scanning electron microscopy. The surface of water kefir grains (Figure 2a) appeared porous and smoother compared with the more heterogeneous surface of milk kefir grains (Figure 2b), which exhibited complex, surface-associated branched structures without evident crystalline assembly, likely consisting of precipitated peptides or other organic materials. No cells were observed directly on the surface, suggesting that they were either encapsulated within the polysaccharide biofilm or removed during sample preparation due to washing.

### 3.2. Removal of Copper(II) Using Water Kefir Grains

From the experimental results of copper(II) sorption onto the biomass of water kefir grains, comprising the microbial cells and their extracellular products, we determined the Freundlich constant (*K_F_*) to be 0.008 (Table 2). Under equilibrium conditions where the sorption system reaches a concentration of 1 mmol∙L^−1^ (Figure 1), this Freundlich constant can be translated into a sorption capacity of water kefir (0.008 mmol∙g^−1^). Since this value specifically applies to diluted solutions, the relatively low sorption capacity indicates a weak affinity of copper(II) for the adsorbent in such diluted systems. Notably, the highest experimentally measured sorption capacity was found to be 0.022 mmol∙g^−1^ (Figure 1), further highlighting the limitations of this biosorbent in retaining copper(II) under the given conditions.

The exponent *N* in the Freundlich isotherm has a value of 1.03 (Table 3), indicating slight variability in the adsorption energies that bind copper(II) from the solution to the surface of the biosorbent, suggesting heterogeneity in the sorption sites.

**Figure 3:**
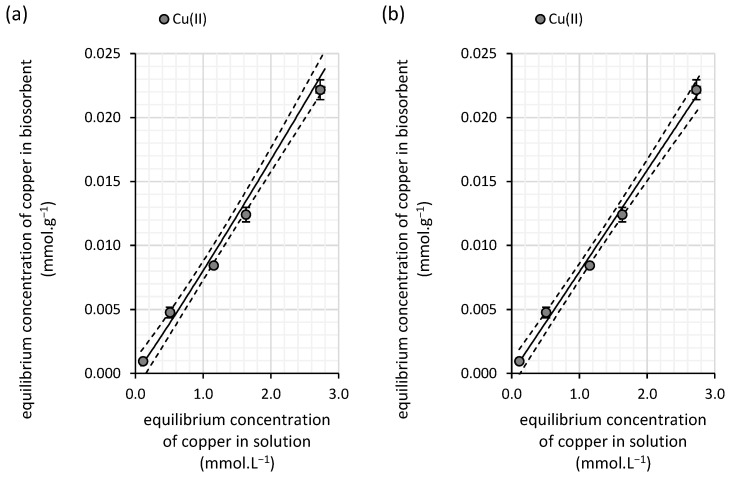
Isotherm fits of the experimental data for copper(II) removal from aqueous solutions by water kefir grains using the (**a**) Freundlich and (**b**) Langmuir isotherm models (solid lines). The experiments were conducted in darkness at 25 °C under dynamic conditions (45 rpm) with initial copper(II) concentrations ranging from 0.17 to 4.0 mmol∙L^−1^ (initial pH of 6.0; contact time of 100 h; n = 3). The dashed line represents the 95% confidence interval for each isotherm model.

The Langmuir model also provides a good fit to the experimentally obtained sorption data with R^2^ reaching 0.99. However, the more statistically sensitive Akaike weight (Table 3) indicates that this model is less suitable for describing the experimental data compared with the Freundlich model. Furthermore, caution is needed when interpreting the Langmuir parameters, particularly due to the model’s behavior and the lack of saturation of the biosorbent across the tested concentration range. The calculated maximum adsorption capacity (*S_max_*) is unrealistic, as the sorption capacity linearly increases with rising equilibrium concentrations and shows no signs of reaching saturation (Figure 3). This discrepancy is further supported by the significantly large error associated with the *S_max_* parameter, which deviates by six orders of magnitude. Therefore, the Langmuir isotherm is not suitable for analyzing copper biosorption by water kefir under the conditions of our study.

**Figure 4:**
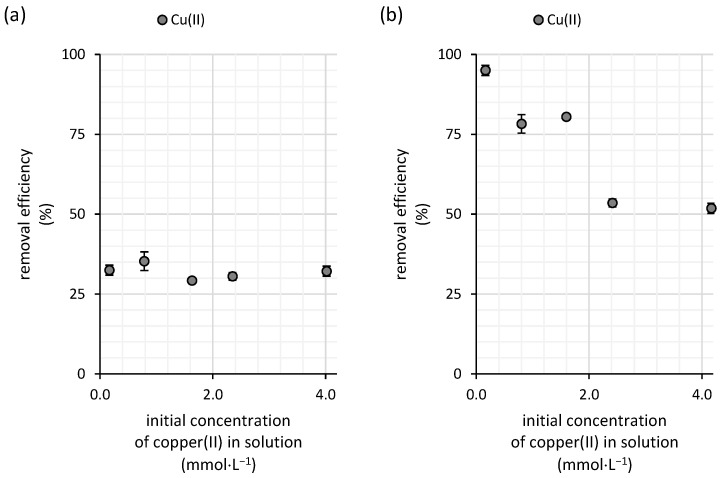
Removal efficiencies for copper(II) from aqueous solutions using (**a**) water and (**b**) milk kefir grains (n = 3; temperature of 25 °C; dynamic conditions (45 rpm); initial copper(II) concentrations ranging from 0.16 to 4.2 mmol∙L^−1^; initial pH of 6.0; contact time of 100 h).

Both the low affinity of copper toward the biomass and the low sorption capacity in diluted solutions, as indicated by the Freundlich constant, contribute to the limited removal efficiency of the biosorbent. As shown in Figure 4, the overall performance of the water kefir biomass in copper(II) removal remains relatively low, with efficiency not exceeding 35.5%. This efficiency shows only minimal variation across different initial copper(II) concentrations, indicating a consistent but limited capacity for copper adsorption.

**Figure 5:**
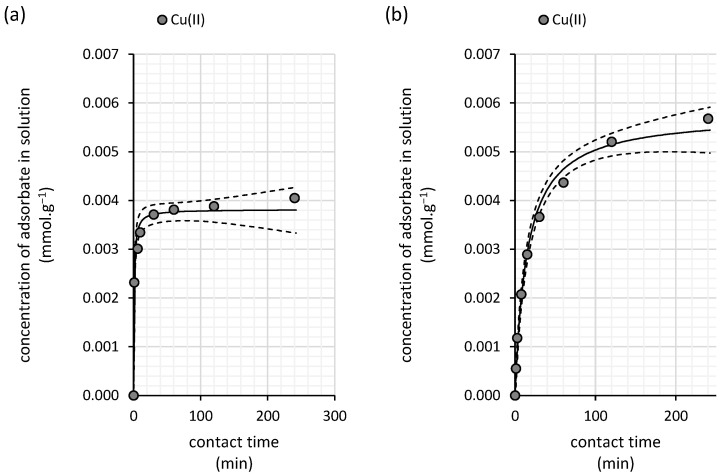
Kinetics of the copper(II) removal from aqueous solutions by (**a**) water kefir and (**b**) milk kefir grains fitted using pseudo-second-order kinetic model (solid line). The experiments were conducted in darkness at 25 °C under dynamic conditions (45 rpm) with initial copper(II) concentration of 0.79 mmol∙L^−1^ (50 mg∙L^−1^; initial pH of 6.0; n = 2). The dashed line represents the 95% confidence interval for the applied kinetic model.

Our kinetic data (Figure 5a) indicate rapid copper(II) removal by water kefir grains, reaching equilibrium after 100 min of contact time. In comparison with the pseudo-first-order model, the experimental data fit better with the pseudo-second-order kinetic model, as suggested by a comparison of Akaike weights, with a rate constant as high as 330.3 g·mmol^−1^·min^−1^ (Table 4).

### 3.3. Removal of Copper(II) Using Milk Kefir Grains

Compared with water kefir grains, the sorption kinetics of copper(II) onto the milk kefir biomass are significantly slower, as indicated by the lower pseudo-second-order kinetic rate constant of only 11.8 g·mmol^−1^·min^−1^ (Table 4). While rapid sorption occurs within the first 100 min, equilibrium is not fully achieved within this period, suggesting a slower, time-dependent phase during which equilibrium may be reached over an extended duration (Figure 5b). Nonetheless, the data fit well with the pseudo-second-order kinetic model (Akaike weight of 0.99) in comparison with the pseudo-first-order kinetic model.

The copper sorption efficiency of the milk kefir biomass declines as the initial copper concentration in the solution increases (Figure 4b). However, at a low copper(II) concentration of 0.17 mmol·L^−1^, milk kefir exhibited a high binding efficiency, removing up to 95% of the copper present. This result demonstrates the strong adsorption capacity of milk kefir in diluted solutions. Even at a concentration of 0.8 mmol·L^−1^, it managed to remove 78% of copper(II). At the highest tested concentration, 4.2 mmol·L^−1^, milk kefir still removed 51% of the copper, showcasing its superior performance compared with water kefir grains (Figure 4b).

The superior effectiveness of milk kefir as a biosorbent for copper removal from aqueous solutions is further supported by the parameters obtained from sorption isotherm analysis (Table 3). The Freundlich constant (*K_F_*) for milk kefir is as high as 0.035, representing a fourfold increase in sorption capacity compared with water kefir at an equilibrium copper concentration of 1 mmol·L^−1^. This higher *K_F_* value suggests a significantly greater affinity of milk kefir for copper(II), particularly in diluted solutions.

**Figure 6:**
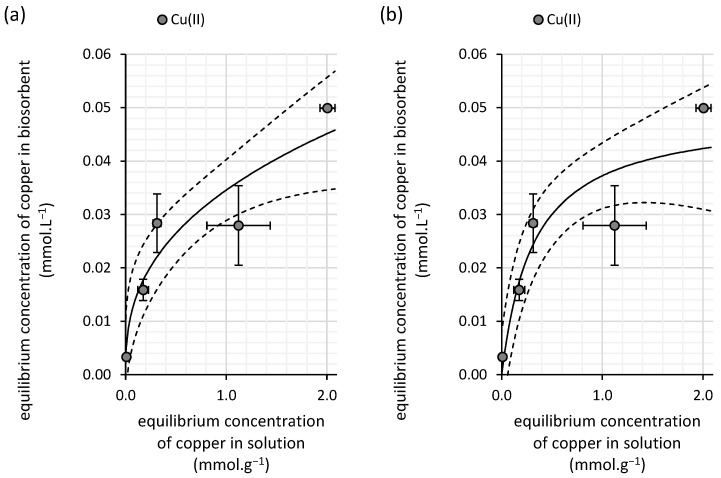
Isotherm fits of the experimental data for copper(II) removal from aqueous solutions by milk kefir grains using the (**a**) Freundlich and (**b**) Langmuir isotherm models (solid lines). The experiments were conducted in darkness at 25 °C under dynamic conditions (45 rpm) with initial copper(II) concentrations ranging from 0.16 to 4.2 mmol∙L^−1^ (initial pH of 6.0; contact time of 100 h; n = 3). The dashed line represents the 95% confidence interval for each isotherm model.

Despite the lower determination coefficient for the Langmuir isotherm model (0.80), the maximum adsorption capacity (*S_max_*) of 0.049 mmol·g^−1^ for milk kefir is more realistic than the value observed for the water kefir biomass. This is reflected in the shape of the isotherm curve, where signs of saturation of the sorbent with copper are evident at the highest tested concentration (Figure 6). These findings suggest that while milk kefir has a notable affinity for copper, its adsorption capacity likely becomes limited at equilibrium concentrations exceeding 2 mmol·L^−1^.

### 3.4. Removal of Arsenic(V) Using Water Kefir Grains

When analyzing the experimental data on arsenic(V) sorption onto the biomass of microorganisms and exopolymers from water kefir using the Langmuir isotherm model, the results showed more realistic parameter estimates compared with copper(II) biosorption. However, the statistical uncertainty remains considerable, as evidenced by the wide error margins for the Langmuir constants: *K_L_* (0.07 ± 0.06 L∙mmol^−1^) and *S_max_* (0.13 ± 0.09 mmol·g^−1^). These substantial errors suggest that the parameters are statistically less significant, necessitating cautious interpretation (Table 5).

**Figure 7:**
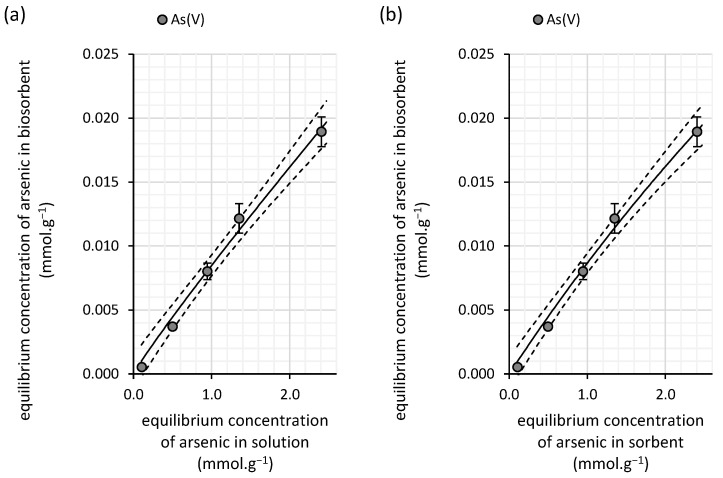
Isotherm fits of the experimental data for arsenic(V) removal from aqueous solutions by water kefir grains using the (**a**) Freundlich and (**b**) Langmuir isotherm models (solid lines). The experiments were conducted in darkness at 25 °C under dynamic conditions (45 rpm) with initial arsenic(V) concentrations ranging from 0.14 to 3.5 mmol∙L^−1^ (initial pH of 6.0; contact time of 100 h; n = 3). The dashed line represents the 95% confidence interval for each isotherm model.

The relationship between sorption capacity and equilibrium arsenic concentration (Figure 7) exhibits a linear trend similar to that observed in copper(II) sorption onto water kefir grains (Figure 6). This suggests that the Freundlich model may provide a more accurate description of the experimental data. This assumption is further supported by the significantly lower statistical error associated with the Freundlich constant *K_F_* (0.008 ± 0.0004) (Table 5), indicating that the sorbent’s affinity for arsenic(V) in diluted solutions is relatively low. Additionally, the exponent *N* takes a value of 0.93, which points to greater homogeneity of the adsorption sites on the surface of the water kefir biomass. This implies a more uniform binding of arsenic(V) from aqueous solutions compared with the binding of copper(II).

**Figure 8:**
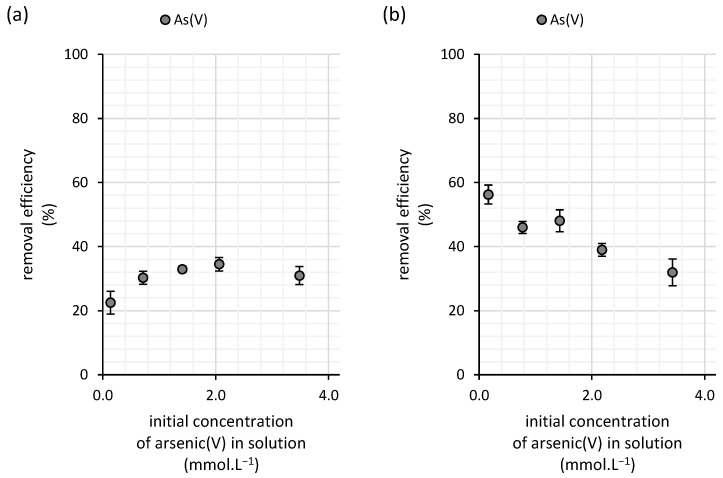
Removal efficiencies for arsenic(V) from aqueous solutions using (**a**) water and (**b**) milk kefir grains (n = 3; temperature of 25 °C; dynamic conditions (45 rpm); initial arsenic(V) concentrations ranging from 0.14 to 3.5 mmol∙L^−1^; initial pH of 6.0; contact time of 100 h).

The sorption efficiency of the water kefir biomass for arsenic(V) varied depending on the concentration of arsenic in the solution, though the overall trend remained relatively stable (Figure 8a). At an initial arsenic concentration of 0.16 mmol·L^−1^, the sorption efficiency was 22.5%. As the arsenic concentration increased to 0.7 mmol·L^−1^, the sorption efficiency rose to 30%, indicating an increased uptake of arsenic by the water kefir biomass. This upward trend continued as the arsenic concentration increased further, reaching a maximum efficiency of 34.5% at an initial concentration of 2.1 mmol·L^−1^. However, beyond this point, sorption efficiency declined to 31%, suggesting that the biosorbent began to approach saturation. This decline indicates that at higher concentrations, the sorbent’s capacity to adsorb additional arsenic diminishes, likely due to the saturation of available adsorption sites.

**Figure 9:**
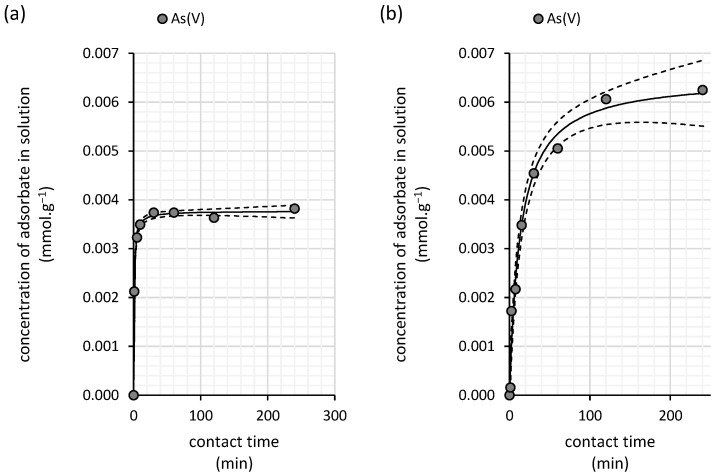
Kinetics of the arsenic(V) removal from aqueous solutions by (**a**) water kefir and (**b**) milk kefir grains fitted using pseudo-second-order kinetic model (solid line). The experiments were conducted in darkness at 25 °C under dynamic conditions (45 rpm) with initial arsenic(V) concentration of 0.67 mmol∙L^−1^ (50 mg∙L^−1^; initial pH of 6.0; n = 2). The dashed line represents the 95% confidence interval for the applied kinetic model.

The water kefir grains exhibit relatively rapid adsorption kinetic for arsenic(V) removal from aqueous solutions, achieving equilibrium in as little as 30 min (Figure 9a). As the pseudo-second-order kinetic model accurately describes the experimental data over a 240 min timeframe (Akaike weight of 0.99), the rate constant derived from this model has a relatively small margin of error, with a value of 338.0 ± 23.7 mmol^−1^·min^−1^ (Table 6).

### 3.5. Removal of Arsenic(V) Using Milk Kefir Grains

Although the changes in milk kefir’s sorption capacity relative to the equilibrium concentration of arsenic are better described by the Langmuir isotherm according to the Akaike weight, we still recommend interpreting this model with caution. This is due to the relatively large error associated with the Langmuir constant *K_L_* (0.78 ± 0.22), as shown in Table 5. In contrast, the Freundlich isotherm presents a much smaller statistical error, suggesting that its parameters are more reliable for describing the experimental data. The Freundlich constant *K_F_*, which reflects the affinity of milk kefir for arsenic, indicates a moderate sorption capacity (0.013 mmol·g^−1^), yet this value is almost twice as high as that for the water kefir biomass (Table 5), reinforcing milk kefir’s superior biosorption efficiency for arsenic(V) removal.

**Figure 10:**
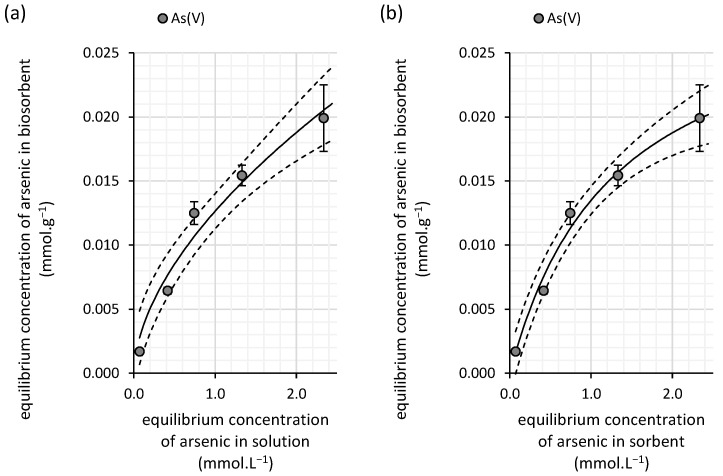
Fits of the experimental data for arsenic(V) removal from aqueous solutions by milk kefir grains using the (**a**) Freundlich and (**b**) Langmuir isotherm models (solid lines). The experiments were conducted in darkness at 25 °C under dynamic conditions (45 rpm) with initial arsenic(V) concentrations ranging from 0.16 to 3.4 mmol∙L^−1^ (initial pH of 6.0; contact time of 100 h; n = 3). The dashed line represents the 95% confidence interval for each isotherm model.

The sorption efficiency of milk kefir exhibits a decreasing linear trend with increasing initial concentrations of arsenic(V) in the solution (Figure 8b). At lower concentrations, the sorption efficiency reached as high as 56%, whereas at the highest tested concentration of 3.4 mmol·L^−1^, the efficiency dropped to 32%, which is only slightly higher than the efficiency observed for water kefir grains (31%) (Figure 8a). This declining trend suggests that milk kefir grain’s sorption capacity is nearing saturation as arsenic concentrations increase, as indicated by the data in Figure 10.

The sorption kinetics of arsenic(V) onto the milk kefir biomass are well described by the pseudo-second-order kinetic model (Table 6), requiring a longer period to reach equilibrium compared with water kefir grains. Equilibrium is achieved more gradually, with a rate constant of 11.7 mmol^−1^·min^−1^, over approximately 240 min (Figure 9b).

## 4. Discussion

Our study demonstrates that water kefir grains generally exhibit lower sorption capacity and overall efficiency compared with the milk kefir biomass (Table 3 and Table 5), particularly in diluted solutions. This difference is likely attributable to variations in the chemical composition and the availability of high-affinity sorption sites responsible for binding copper(II) and arsenic(V) from aqueous solutions.

The primary organic exopolymers in water kefir grains consist of insoluble O3- and O2-branched dextrans, along with smaller quantities of levans [15]. Both of these polysaccharides contain numerous hydroxyl (-OH) groups [20], which act as key sorption sites, enabling interactions with metals and metalloids, thereby facilitating their binding.

The results of our FTIR analysis of both kefir biomasses (Figure 1) support the presence of these heavy-metal reactive functional groups of polysaccharides. The fingerprint region between 1200 and 900 cm^−1^ highlights the presence of pyranose rings in kefir grains, with glucose present in water kefir and both glucose and galactose in milk kefir [21]. In contrast to water kefir, the matrix of milk kefir grains incorporates not only polysaccharides but also a significant amount of proteins [3], which can contribute up to 29% of the dry grain mass [22]. This composition enhances the diversity of sorption sites by introducing functional groups such as amino (-NH_2_). Both arsenic(V) and copper(II) exhibit affinities for these extracellular polymeric substances [23,24]. Thus, it is likely that the protein-associated sorption sites within the milk kefir grains provide high-affinity binding locations for both arsenic(V) and copper(II), significantly enhancing the sorption performance of milk kefir grains in their removal from aqueous solutions.

The results of the FTIR analysis by Naveed, et al. [25] suggest the direct involvement of -OH, -NH-, -CO-, and C=O groups from extracellular polymeric substances in arsenic sorption, as evidenced by band shifts following arsenic(V) exposure in circumneutral solutions. In our experiment, the initial pH of the arsenic(V) solution was adjusted to 6.0, so the primary functional groups in the kefir polysaccharide matrix responsible for arsenic(V) and copper(II) removal are expected to remain negatively charged. This is consistent with findings on kefiran, which is only positively charged below pH 2 [26]—a typical trait of bacterial extracellular polymeric substances [27]. This negative charge may have impeded arsenic(V) adsorption due to repulsive interactions with the kefir grain surfaces, given that arsenic(V) predominantly exists as the negatively charged H_2_AsO_4_^-^ species within our experimental pH range under oxidizing conditions, as indicated by its behavior in natural waters [28]. Conversely, copper(II) remains stable in cationic form, and observed shifts toward more acidic regions during kinetic and isotherm studies suggest a strong interaction with the negatively charged surfaces of kefir grains. This interaction likely involves the formation of inner-sphere complexes with oxygen-bearing ligands, e.g., carboxyl groups that are typically considered the primary sorption sites for copper(II) in bacterial extracellular polymeric matrices [29]. Arsenic, on the other hand, has been reported to strongly interact with amino groups in proteins, such as tyrosine- and tryptophan-like proteins within the extracellular polymeric substances of *Chlorella* sp. [30]. Thus, we hypothesize that the presence of amino groups of proteins, along with other functional groups such as hydroxyl, likely contributes to this improved efficiency, as these sites facilitate stronger interactions with the metal ions and metalloids. In contrast, water kefir’s polysaccharide-based matrix offers fewer high-affinity sites, leading to its comparatively lower performance in arsenic(V) and copper(II) removal in diluted solutions (Figure 4 and Figure 8).

The differences in sorption sites, their distribution, and their impact on the sorption mechanism can also be inferred from the isotherm analysis. For both copper(II) and arsenic(V), saturation is observed in the case of milk kefir (Figure 6 and Figure 10), suggesting a stronger affinity but a lower density of high-affinity sorption sites within the milk grains. In contrast, water kefir grains align more closely with the Freundlich isotherm (Figure 3 and Figure 7), indicating lower affinity but a relatively high frequency of sorption sites. This suggests that while water kefir grains possess numerous sorption sites, these are less selective for copper(II) and arsenic(V) compared with milk kefir. Furthermore, the sorption pattern observed for water kefir aligns with a C-type isotherm, indicating preferential removal through the micropores of the grains or possibly due to a more pronounced breakdown of the polymeric matrix within water kefir grains [31]. The results of our SEM analysis (Figure 2) highlighted notable structural and morphological differences between the two types of kefir grains. Water kefir grains exhibit a porous, hydrogel-like morphology, whereas milk kefir grains display a more compact surface populated with clustered, branched organic structures. It is likely that, due to more compact and structurally stable nature of milk kefir grains, sorption occurs primarily through more specific binding mechanisms rather than through porous absorption. However, differences in the sorption mechanisms are more precisely inferred from the kinetic studies. Interestingly, regardless of the type of kefir grain or element, all data fit the pseudo-second-order kinetic model exceptionally well. Conversely, the goodness of fit of the pseudo-first-order kinetic model is very low (Table 4 and Table 6). This allows us to assume certain considerations that can be asserted regarding the suitability of fitting the pseudo-second-order kinetic model. These include the following: (i) the adsorption process involves chemisorption, and (ii) the rate-limiting process is the chemical reaction step [32]. Nevertheless, as indicated by the comparison of rate constants, equilibrium is achieved significantly faster with water kefir (Figure 5 and Figure 9), suggesting that particle boundary diffusion, whether through pore or solid diffusion, may play a more prominent role in the adsorption process of both arsenic(V) and copper(II) onto the kefir biomass.

Based on the analyzed data, the milk kefir biomass emerges as a more effective biosorbent for arsenic at lower concentrations compared with the water kefir biomass. Unfortunately, to the best of our knowledge, no systematic or experimental studies have explored the potential of milk kefir grains for removing other heavy metals from solutions. Nevertheless, water kefir grains demonstrate relatively stable removal performance across various cationic heavy metals, including copper(II), nickel(II), and lead(II), as reported by Volpi, et al. [5]. This stability highlights their potential applicability for multicomponent adsorption in real industrial effluents or contaminated surface and groundwater. Still, to gain a clearer perspective, it is essential to compare water and milk kefir grains’ performance with other unmodified waste biosorbents to properly assess its sorptive efficiency and potential for practical applications. Table 7 illustrates that the maximum sorption capacity derived from the Langmuir model is moderate and comparable with other non-modified waste-biomass-based biosorbents. However, the sorption capacity of chemically and physically modified biomasses far exceeds that of milk kefir, with modified materials often capable of removing hundreds of micrograms of arsenate per gram. This highlights the potential for significant improvements to enhance kefir’s competitive edge. While some modification techniques still result in comparable performance, such as iron-coated cork granulates with a maximum sorption capacity of 56.07 µmol·g^−1^ [33] or wood-derived biochar with 51.9 µmol·g^−1^ [34], other approaches, particularly thermal treatments combined with nanosized inorganic compounds, yield much higher capacities. For example, zero-valent iron-composited palm-waste biochars reach a sorption capacity of 354 µmol·g^−1^ [35], and corncob-derived biochar impregnated with ZnO achieves 346 µmol·g^−1^ [36]. These modifications significantly outperform the untreated kefir grains, suggesting that targeted enhancements to kefir biosorbents can greatly improve their effectiveness for metal removal.

The performance of kefir grains in our study was notably lower compared with other available data on the sorptive removal of copper(II) from aqueous solutions using non-modified waste biomasses (Table 5). This discrepancy highlights the limitations of kefir grains as biosorbents in practical applications, particularly for copper removal, suggesting that kefir grains would require significant modification or enhancement to compete with other well-established biosorbents. This enhancement can be achieved through either chemical or physical modification of kefir grains or a combination of both approaches. For instance, processing the waste biomass into activated carbons can significantly improve the heavy-metal removal capacity by increasing the surface area, optimizing the pore distribution, and enhancing the chemical nature of the active sorption sites on biochar-based adsorbents [44,45].

Recently, nanobiocomposites have emerged as a promising and highly effective strategy for removing toxic metals and metalloids from contaminated aqueous environments. By combining the natural sorption properties of biological materials with the enhanced functional capabilities of nanoparticles, these composites deliver superior performance [46]. However, despite their potential, the reported effectiveness of nanobiocomposites for copper removal significantly varies. In some cases, they do not necessarily outperform untreated waste materials [47]. Nevertheless, while the removal efficiency of copper(II) by kefir grains shows potential in diluted copper solutions (Figure 4b), their overall sorption capacity remains underwhelming (Table 8). This limitation significantly reduces their practical applicability in real-world scenarios, particularly when compared with more eco-friendly alternatives like nanobiocomposites, which have demonstrated superior performance relative to the sorption capacity of kefir grains [48].

## 5. Conclusions

In this study, we investigated the efficiency of water and milk kefir grains as biosorbents for the removal of copper(II) and arsenic(V) from aqueous solutions. Our results revealed a stark contrast in performance between the two types of kefir grains. Notably, milk kefir exhibited a remarkable capacity to remove up to 95% of copper(II) at a low concentration of 0.16 mmol·L^−1^ and achieved a maximum removal efficiency of 56% for arsenic(V) at lower concentrations. This demonstrates its strong adsorption capabilities, especially in diluted solutions. In contrast, water kefir grains achieved a maximum removal efficiency of only 35.5% for copper(II) and 34.5% for arsenic(V), highlighting their limited effectiveness as a biosorbent for these contaminants.

Isotherm analyses further confirmed the superior performance of milk kefir. The results of the analysis indicated that water kefir has a lower affinity for copper and arsenic, which can be attributed to its simpler polysaccharide composition and the limited availability of high-affinity sorption sites. In contrast, milk kefir’s more complex matrix, enriched with proteins and diverse functional groups, significantly enhances its binding capabilities for metal ions.

Despite the promising results, it is crucial to recognize that the overall performance of kefir grains remains lower when compared with other modified biosorbents and nanobiocomposites, which have consistently demonstrated superior metal removal efficiencies. This underscores the necessity for further modifications to the kefir biomass to enhance its sorption capacity and make it more competitive in practical applications. Future research should focus on optimizing the chemical and structural properties of kefir grains to improve their performance in real-world scenarios, thereby contributing to more sustainable and effective methods for remediating contaminated water sources.

## Figures and Tables

**Table 1:** The isotherm models used to evaluate the sorptive performance of the water and milk kefir grains.

Isotherm	Equation	Constants
Langmuir	$S_{eq}=\frac{S_{max}K_{L}C_{eq}}{1+K_{L}C_{eq}}$	*S_max_* (mmol·g^−1^) is the theoretical sorption capacity representing the maximum amount of adsorbate that can be adsorbed per unit mass of the sorbent, and *K_L_* (L·mmol^−1^) is the Langmuir sorption constant, which indicates the affinity of the adsorbate (arsenic or copper) toward the sorbent
Freundlich	$S_{eq}=K_{F}C_{eq}^{N}$	*N* is an empirical parameter related to the intensity of sorption, and *K_F_* (mmol^1-N^·L^N^·g^−1^) is the Freundlich constant that represents the sorption capacity at a unitary equilibrium concentration of adsorbate (arsenic or copper) in the solution

**Table 2:** The models for the reaction kinetics used to evaluate the sorptive performance of the water and milk kefir grains.

Kinetic Model	Equation	Constants
Pseudo-first order	$S_{t}=S_{eq}\;(1-e^{-k_{1}t})$	*k*_1_ (min^−1^) represents the rate constants for the pseudo-first-order kinetics
Pseudo-second order	$S_{t}=\frac{{k_{2}S}_{eq}^{2}t}{1+k_{2}S_{eq}t\;}\;\;\;\;\;\;\;\;\;$	*k*_2_ (g·mmol^−1^·min^−1^) represents the rate constants for the pseudo-second-order kinetics

**Table 3:** Langmuir and Freundlich isotherm parameters for the sorption of copper(II) using water and milk kefir grains (n = 3; temperature of 25 °C; dynamic conditions (45 rpm); initial copper(II) concentrations ranging from 0.16 to 4.2 mmol∙L^−1^; initial pH of 6.0; contact time of 100 h).

**Langmuir Isotherm**	**Culture**	***K_L_*** **(L·mmol^−1^)**	***S_max_*** **(mmol·g^−1^)**	**R^2^**	**Akaike weight**
	Water kefir	5.32·10^−6^ ± 0.03·10^0^	1.5·10^3^ ± 8.5·10^6^	0.99	0.44
	Milk kefir	3.2 ± 1.7	0.049 ± 0.008	0.80	0.32
**Freundlich Isotherm**	**Culture**	***K_F_*** **(mmol^1-N^·L^N^·g^−1^)**	***N***	**R^2^**	**Akaike weight**
	Water kefir	0.008 ± 0.0003	1.03 ± 0.05	0.99	0.56
	Milk kefir	0.035 ± 0.003	0.38 ± 0.09	0.83	0.68

**Table 4:** Kinetic model parameters for the sorption of copper(II) using water and milk kefir grains (n = 2; temperature of 25 °C; dynamic conditions (45 rpm); initial copper(II) concentrations of 0.79 mmol∙L^−1^; initial pH of 6.0).

**Pseudo-first-order kinetic model**	**Culture**	***S_eq_*** **(mmol·g^−1^)**	***k*_1_** **(min^−1^)**	**R^2^**	**Akaike weight**
	Water kefir	0.0036 ± 0.0001	0.995 ± 0.269	0.94	0.02
	Milk kefir	0.0051 ± 0.0003	0.053 ± 0.009	0.96	0.01
**Pseudo-second-order kinetic model**	**Culture**	***S_eq_*** **(mmol·g^−1^)**	***k*_2_** **(g·mmol^−1^·min^−1^)**	**R^2^**	**Akaike weight**
	Water kefir	0.0038 ± 0.0001	330.3 ± 82.1	0.98	0.98
	Milk kefir	0.0058 ± 0.0002	11.8 ± 1.83	0.99	0.99

**Table 5:** Langmuir and Freundlich isotherm parameters for the sorption of arsenic(V) using water and milk kefir biomasses (n = 3; temperature of 25 °C; dynamic conditions (45 rpm); initial arsenic(V) concentrations ranging from 0.14 to 3.5 mmol∙L^−1^; initial pH of 6.0; contact time of 100 h).

**Langmuir isotherm**	**Culture**	***K_L_*** **(L·mmol^−1^)**	***S_max_*** **(mmol·g^−1^)**	**R^2^**	**Akaike weight**
	Water kefir	0.07 ± 0.06	0.13 ± 0.09	0.98	0.63
	Milk kefir	0.78 ± 0.22	0.03 ± 0.004	0.96	0.91
**Freundlich isotherm**	**Culture**	***K_F_*** **(mmol^1-N^·L^N^·g^−1^)**	***N***	**R^2^**	**Akaike weight**
	Water kefir	0.008 ± 0.0004	0.93 ± 0.07	0.98	0.37
	Milk kefir	0.013 ± 0.0007	0.57 ± 0.07	0.93	0.09

**Table 6:** Kinetic model parameters for the sorption of arsenic(V) using water and milk kefir grains (n = 2; temperature of 25 °C; dynamic conditions (45 rpm); initial arsenic(V) concentrations of 0.67 mmol∙L^−1^; initial pH of 6.0).

**Pseudo-first-order kinetic model**	**Culture**	***S_eq_*** **(mmol·g^−1^)**	***k*_1_** **(min^−1^)**	**R^2^**	**Akaike weight**
	Water kefir	0.0036 ± 0.0001	0.843 ± 0.121	0.98	0.01
	Milk kefir	0.0058 ± 0.0003	0.059 ± 0.011	0.97	0.04
**Pseudo-second-order kinetic model**	**Culture**	***S_eq_*** **(mmol·g^−1^)**	***k*_2_** **(g·mmol^−1^·min^−1^)**	**R^2^**	**Akaike weight**
	Water kefir	0.0038 ± 0.0001	338.0 ± 23.7	0.99	0.99
	Milk kefir	0.0065 ± 0.0003	11.7 ± 2.29	0.98	0.96

**Table 7:** Comparison of maximum sorption capacities of waste-biomass-based sorbents derived from the Langmuir isotherm (*S_max_*) for arsenic(V).

Type of Sorbent	*S_max_* (µmol·g^−1^)	Reference
Milk kefir grains	30	This work
Fish scale	0.38	Rahaman, et al. [37]
Wheat straw	1.5	Ebrahimi, et al. [38]
Rice polish	1.96	Ranjan, et al. [39]
Anaerobic sludge	2.19	Chowdhury and Mulligan [40]
Tea waste	28.3	Kamsonlian, et al. [41]
Sawdust	58.74	Leal, et al. [42]
Almond shell	141.7	Ali, et al. [43]

**Table 8:** Comparison of maximum sorption capacities of waste-biomass-based sorbents derived from the Langmuir isotherm (*S_max_*) for copper(II).

Type of Sorbent	*S_max_* (µmol·g^−1^)	Reference
Milk kefir grains	49	This work
Pine sawdust	35	Orozco, et al. [49]
Activated sludge biomass	138	Aslan, et al. [50]
Rice straw	196	Buasri, et al. [51]
Cone biomass of *Thuja orientalis*	303	Nuhoglu and Oguz [52]
Coffee grounds	398	Młynarczykowska and Orlof-Naturalna [53]
Sugarcane bagasse	841	Shah, et al. [54]

## Data Availability

The original contributions presented in this study are included in the article. Further inquiries can be directed to the corresponding author.

## References

[1] Karić N., Maia A.S., Teodorović A., Atanasova N., Langergraber G., Crini G., Ribeiro A.R.L., Đolić M. (2022). Bio-waste valorisation: Agricultural wastes as biosorbents for removal of (in)organic pollutants in wastewater treatment. Chem. Eng. J. Adv..

[2] Karnwal A. (2024). Unveiling the promise of biosorption for heavy metal removal from water sources. Desalination Water Treat..

[3] Gao X., Li B. (2016). Chemical and microbiological characteristics of kefir grains and their fermented dairy products: A review. Cogent Food Agric..

[4] Asadi Touranlou F., Noori S.M.A., Salari A., Afshari A., Hashemi M. (2023). Application of kefir for reduction of contaminants in the food industry: A systematic review. Int. Dairy J..

[5] Volpi G., Ginepro M., Tafur-Marinos J., Zelano V. (2019). Pollution Abatement of Heavy Metals in Different Conditions by Water Kefir Grains as a Protective Tool against Toxicity. J. Chem..

[6] Li Y., Qi X., Li G., Wang H. (2021). Efficient removal of arsenic from copper smelting wastewater via a synergy of steel-making slag and KMnO4. J. Clean. Prod..

[7] Rehman M., Liu L., Wang Q., Saleem M.H., Bashir S., Ullah S., Peng D. (2019). Copper environmental toxicology, recent advances, and future outlook: A review. Environ. Sci. Pollut. Res..

[8] Hughes M.F., Beck B.D., Chen Y., Lewis A.S., Thomas D.J. (2011). Arsenic Exposure and Toxicology: A Historical Perspective. Toxicol. Sci..

[9] Hagarová I. (2007). Speciation of arsenic in waters by AAS techniques. Chem. Listy.

[10] Hagarová I., Kubová J. (2008). Speciation of antimony in waters using separation coupled with atomic spectrometry. Chem. Listy.

[11] Hagarová I., Nemček L., Lichtfouse E. (2021). Recent advances in speciation analysis of trace antimony in environmental and biological samples based on cloud point extraction and spectrometric methods. Sustainable Agriculture Reviews.

[12] Bozdogan H. (1987). Model selection and Akaike’s Information Criterion (AIC): The general theory and its analytical extensions. Psychometrika.

[13] Piermaria J., Bosch A., Pinotti A., Yantorno O., Garcia M.A., Abraham A.G. (2011). Kefiran films plasticized with sugars and polyols: Water vapor barrier and mechanical properties in relation to their microstructure analyzed by ATR/FT-IR spectroscopy. Food Hydrocoll..

[14] Ramírez Tapias Y.A., Rezzani G.D., Delgado J.F., Peltzer M.A., Salvay A.G. (2024). New Materials from the Integral Milk Kefir Grain Biomass and the Purified Kefiran: The Role of Glycerol Content on the Film’s Properties. Polymers.

[15] Fels L., Jakob F., Vogel R.F., Wefers D. (2018). Structural characterization of the exopolysaccharides from water kefir. Carbohydr. Polym..

[16] Dziuba B., Babuchowski A., Nałęcz D., Niklewicz M. (2007). Identification of lactic acid bacteria using FTIR spectroscopy and cluster analysis. Int. Dairy J..

[17] Rutten M.J.M., Bovenhuis H., Hettinga K.A., van Valenberg H.J.F., van Arendonk J.A.M. (2009). Predicting bovine milk fat composition using infrared spectroscopy based on milk samples collected in winter and summer. J. Dairy Sci..

[18] Alves E., Ntungwe E.N., Gregório J., Rodrigues L.M., Pereira-Leite C., Caleja C., Pereira E., Barros L., Aguilar-Vilas M.V., Rosado C. (2021). Characterization of Kefir Produced in Household Conditions: Physicochemical and Nutritional Profile, and Storage Stability. Foods.

[19] Zhang L., Liu J., Kong S., Chen N., Hung W.-L., Zhao W., Zeng Z., Zhang J., Yang Z. (2023). Lipoteichoic acid obtained from Lactobacillus paracasei via low-temperature pasteurization alleviates the macrophage inflammatory response by downregulating the NF-κB signaling pathway. J. Funct. Foods.

[20] Coma M.E., Peltzer M.A., Delgado J.F., Salvay A.G. (2019). Water kefir grains as an innovative source of materials: Study of plasticiser content on film properties. Eur. Polym. J..

[21] Prado M.R., Blandón L.M., Vandenberghe L.P.S., Rodrigues C., Castro G.R., Thomaz-Soccol V., Soccol C.R. (2015). Milk kefir: Composition, microbial cultures, biological activities, and related products. Front. Microbiol..

[22] Han X., Yi H., Zhao S., Sun J., Wang Y. (2020). Prospects of Artificial Kefir Grains Prepared by Cheese and Encapsulated Vectors to Mimic Natural Kefir Grains. J. Food Qual..

[23] Naveed S., Li C., Zhang J., Zhang C., Ge Y. (2020). Sorption and transformation of arsenic by extracellular polymeric substances extracted from *Synechocystis* sp. PCC6803. Ecotoxicol. Environ. Saf..

[24] Zanetti R., Zecchin S., Colombo M., Borgonovo G., Mazzini S., Scaglioni L., Facchetti G., Gandolfi R., Rimoldi I., Cavalca L. (2022). N^i2+^ and Cu^2+^ Biosorption by EPS-Producing Serratia plymuthica Strains and Potential Bio-Catalysis of the Organo–Metal Complexes. Water.

[25] Naveed S., Yu Q., Zhang C., Ge Y. (2020). Extracellular polymeric substances alter cell surface properties, toxicity, and accumulation of arsenic in Synechocystis PCC6803. Environ. Pollut..

[26] Exarhopoulos S., Raphaelides S.N., Kontominas M.G. (2018). Conformational studies and molecular characterization of the polysaccharide kefiran. Food Hydrocoll..

[27] Yang L., Chen Z., Zhang Y., Lu F., Liu Y., Cao M., He N. (2023). Hyperproduction of extracellular polymeric substance in Pseudomonas fluorescens for efficient chromium (VI) absorption. Bioresour. Bioprocess..

[28] Smedley P.L., Kinniburgh D.G. (2002). A review of the source, behaviour and distribution of arsenic in natural waters. Appl. Geochem..

[29] Fang L., Yang S., Huang Q., Xue A., Cai P. (2014). Biosorption mechanisms of Cu(II) by extracellular polymeric substances from Bacillus subtilis. Chem. Geol..

[30] Zhang J., Zhou F., Liu Y., Huang F., Zhang C. (2020). Effect of extracellular polymeric substances on arsenic accumulation in Chlorella pyrenoidosa. Sci. Total Environ..

[31] Giles C.H., Smith D., Huitson A. (1974). A general treatment and classification of the solute adsorption isotherm. I. Theoretical. J. Colloid Interface Sci..

[32] Ho Y.S., McKay G. (1999). Pseudo-second order model for sorption processes. Process Biochem..

[33] Carneiro M.A., Pintor A.M.A., Boaventura R.A.R., Botelho C.M.S. (2022). Efficient removal of arsenic from aqueous solution by continuous adsorption onto iron-coated cork granulates. J. Hazard. Mater..

[34] Niazi N.K., Bibi I., Shahid M., Ok Y.S., Shaheen S.M., Rinklebe J., Wang H., Murtaza B., Islam E., Farrakh Nawaz M. (2018). Arsenic removal by Japanese oak wood biochar in aqueous solutions and well water: Investigating arsenic fate using integrated spectroscopic and microscopic techniques. Sci. Total Environ..

[35] Ahmad M., Usman A.R.A., Hussain Q., Al-Farraj A.S.F., Tsang Y.F., Bundschuh J., Al-Wabel M.I. (2020). Fabrication and evaluation of silica embedded and zerovalent iron composited biochars for arsenate removal from water. Environ. Pollut..

[36] Cruz G.J.F., Mondal D., Rimaycuna J., Soukup K., Gómez M.M., Solis J.L., Lang J. (2020). Agrowaste derived biochars impregnated with ZnO for removal of arsenic and lead in water. J. Environ. Chem. Eng..

[37] Rahaman M.S., Basu A., Islam M.R. (2008). The removal of As(III) and As(V) from aqueous solutions by waste materials. Bioresour. Technol..

[38] Ebrahimi R., Maleki A., Shahmoradi B., Daraei H., Mahvi A.H., Barati A.H., Eslami A. (2013). Elimination of arsenic contamination from water using chemically modified wheat straw. Desalination Water Treat..

[39] Ranjan D., Talat M., Hasan S.H. (2009). Biosorption of arsenic from aqueous solution using agricultural residue ‘rice polish’. J. Hazard. Mater..

[40] Chowdhury M.R.I., Mulligan C.N. (2011). Biosorption of arsenic from contaminated water by anaerobic biomass. J. Hazard. Mater..

[41] Kamsonlian S., Suresh S., Majumder C.B., Chand S. (2011). Biosorption Of As(V) From Contaminated Water Onto Tea Waste Biomass: Sorption Parameters Optimization, Equilibrium and Thermodynamic Studies. J. Future Eng. Technol..

[42] Leal M.A.L., Martínez R.C., Villanueva R.A.C., Flores H.E.M., Penagos C.D.C. (2012). Arsenate Biosorption by Iron-Modified Pine Sawdust in Batch Systems: Kinetics and Equilibrium Studies. Bioresources.

[43] Ali S., Rizwan M., Shakoor M.B., Jilani A., Anjum R. (2020). High sorption efficiency for As(III) and As(V) from aqueous solutions using novel almond shell biochar. Chemosphere.

[44] Demirbas E., Dizge N., Sulak M.T., Kobya M. (2009). Adsorption kinetics and equilibrium of copper from aqueous solutions using hazelnut shell activated carbon. Chem. Eng. J..

[45] Demiral H., Güngör C. (2016). Adsorption of copper(II) from aqueous solutions on activated carbon prepared from grape bagasse. J. Clean. Prod..

[46] Singh S., Kumar V., Datta S., Dhanjal D.S., Sharma K., Samuel J., Singh J. (2020). Current advancement and future prospect of biosorbents for bioremediation. Sci. Total Environ..

[47] Kayalvizhi K., Alhaji N.M.I., Saravanakkumar D., Mohamed S.B., Kaviyarasu K., Ayeshamariam A., Al-Mohaimeed A.M., AbdelGawwad M.R., Elshikh M.S. (2022). Adsorption of copper and nickel by using sawdust chitosan nanocomposite beads—A kinetic and thermodynamic study. Environ. Res..

[48] Eldeeb T.M., El-Nemr A., Khedr M.H., El-Dek S.I. (2021). Novel bio-nanocomposite for efficient copper removal. Egypt. J. Aquat. Res..

[49] Orozco C.I., Freire M.S., Gómez-Díaz D., González-Álvarez J. (2023). Removal of copper from aqueous solutions by biosorption onto pine sawdust. Sustain. Chem. Pharm..

[50] Aslan S., Yldiz S., Ozturk M. (2018). Biosorption of Cu^2+^ and Ni^2+^ Ions From Aqueous Solutions Using Waste Dried Activated Sludge Biomass. Pol. J. Chem. Technol..

[51] Buasri A., Chaiyut N., Tapang K., Jaroensin S., Panphrom S. (2012). Removal of Cu^2+^ from Aqueous Solution by Biosorption on Rice Straw—An Agricultural Waste Biomass. Int. J. Environ. Sci. Dev..

[52] Nuhoglu Y., Oguz E. (2003). Removal of copper(II) from aqueous solutions by biosorption on the cone biomass of *Thuja orientalis*. Process Biochem..

[53] Młynarczykowska A., Orlof-Naturalna M. (2024). Biosorption of Copper (II) Ions Using Coffee Grounds—A Case Study. Sustainability.

[54] Shah G.M., Nasir M., Imran M., Bakhat H.F., Rabbani F., Sajjad M., Umer Farooq A.B., Ahmad S., Song L. (2018). Biosorption potential of natural, pyrolysed and acid-assisted pyrolysed sugarcane bagasse for the removal of lead from contaminated water. PeerJ.

